# Knowledge, Attitudes, and Determinants of Nonalcoholic Fatty Liver Disease Among Adults in Jazan Province: A Cross-Sectional Study

**DOI:** 10.7759/cureus.66837

**Published:** 2024-08-14

**Authors:** Ali M Someili, Mostafa Mohrag, Bandar S Rajab, Abdulrahman A Daghreeri, Fawaz M Hakami, Riyadh A Jahlan, Abdulrahman A Otaif, Abdulelah A Otaif, Hussam T Hakami, Bandar F Daghriri, Ameer M Mobarki, Rakan B Almjlad, Mousa Mobarki

**Affiliations:** 1 Internal Medicine and Gastroenterology, Faculty of Medicine, Jazan University, Jazan, SAU; 2 Internal Medicine, Faculty of Medicine, Jazan University, Jazan, SAU; 3 Family and Community Medicine, Jazan University, Jazan, SAU; 4 Medicine, Majmaah University, Al Majma'ah, SAU; 5 Basic Medical Sciences (Pathology), Faculty of Medicine, Jazan University, Jazan, SAU

**Keywords:** saudi arabia, jazan, attitudes, knowledge, nafld

## Abstract

Background

Nonalcoholic fatty liver disease (NAFLD) is the most common liver disease globally, with its prevalence rising worldwide. This study aimed to evaluate the knowledge, attitudes, and determinants related to NAFLD among adults in Jazan, Saudi Arabia.

Methods

A cross-sectional study was conducted using a validated online questionnaire distributed to 540 participants in Jazan Province. Data analysis involved descriptive and comparative statistics to assess knowledge, attitudes, and influencing factors related to NAFLD.

Results

The majority of participants (244, 45.2%) demonstrated poor knowledge about NAFLD, while 226 (41.9%) had fair knowledge. Notably, individuals aged 40-49, males, healthcare workers, those with obesity and diabetes mellitus, and those with a family history of NAFLD showed significantly higher levels of knowledge (p < 0.05). Regarding attitudes, most participants (64.4%) exhibited a positive attitude toward NAFLD, 28.3% had a satisfactory attitude, and only 7% demonstrated a poor attitude.

Conclusion

The findings highlight the need for targeted educational interventions and public awareness campaigns to enhance the general public’s understanding of NAFLD. Providing accurate and up-to-date information about the disease, its consequences, and preventive measures is crucial for improving awareness and knowledge.

## Introduction

Based on many studies, it has been indicated that nonalcoholic fatty liver disease (NAFLD) is the most prevalent liver disease among the global population. NAFLD is a metabolic disease characterized by many characteristics, including obesity, particularly central abdominal fat accumulation. Based on recent studies, it has been indicated that obesity is abundant among NAFLD patients. Furthermore, NAFLD in patients without a history of alcohol consumption can result from the buildup of lipids in the liver, and in most cases, it can impact up to 5% of hepatocytes [1]. NAFLD is a significant contributor to other liver diseases and liver-related mortality. According to a meta-analysis by Musso et al., NAFLD has many associations with cardiovascular mortality, and it can increase the risk of developing type 2 diabetes by twofold [2]. Even more, it can increase the risk of developing chronic kidney diseases [3]. A study by Powell et al. estimated that NAFLD affects up to a quarter of the global population [4]. While advanced liver fibrosis can be one of the most significant indicators of hepatic-related mortality, cardiovascular disease and extrahepatic malignancies are still the leading causes of death for patients with NAFLD [4].

Intricate relationships among several factors contribute to the pathogenesis of the condition. Those factors can involve environmental, demographic, metabolic, and even epigenetic factors. Also, various kinds of gut microbiota can be involved in pathogenesis. It is believed that in recent years, NAFLD can result from excessive lipid buildup, particularly hepatic lipid accumulations, which occur due to the imbalance between fatty acid production and metabolism within the liver. These changes can be a result of other factors, such as changes in lifestyle combined with changes in diet. A low-activity lifestyle and a lipid-rich diet can be two of the significant causes of such changes. Other risk factors, such as smoking and inhaling polluted air, or even the existence of other diseases such as dyslipidemia and type 2 diabetes, can significantly increase the risk of developing NAFLD in combination with the other risk factors mentioned earlier [1,5].

According to recent studies, the prevalence of NAFLD among the global population can be estimated at an average of 25% [5]. The prevalence differs for different ethnicities within the same population, as it is estimated to be lower for African Americans in comparison to Hispanics. Some countries have been reported to have a higher prevalence as well in comparison to the global population, such as the Middle East and South America, where they reported a prevalence of 30-32%, respectively [5]. A systemic review and meta-analysis that evaluated the prevalence of NAFLD in Saudi Arabia indicated that the prevalence of NAFLD was 16.7% (95% CI: 11.1-22.5) [6].

In terms of evaluating the knowledge of NAFLD among different populations, several studies indicated gaps and misconceptions about the disease, which were significant. Based on a study conducted in Hong Kong, it was reported that most of the population had no or little awareness of the condition. Almost half of the population had no awareness of the prevalence and was unclear about the diagnosis. Moreover, more than two-thirds (78%) of participants had no idea about what diagnostic tests and tools are required to diagnose the condition [7]. Similarly, a study conducted in Malaysia reported that less than one-third of the participants were aware of the risk factors associated with the condition, as well as the screening methods and consequences associated with NAFLD [8]. In an equivalent way, a study published in the United States reported that most of the population had no awareness of NAFLD [9]. At the local level, a study conducted in Taif, Saudi Arabia, reported that 63.4% of the participants were unaware of NAFLD, and misconceptions about the disease were present. However, after discussion about the condition, most of the participants had a good perception and attitude toward the condition [10].

As stated in the literature, NAFLD is the most prevalent liver disease in the global population. Its prevalence is increasing every year, and it has been predicted that it can be the leading cause of liver cirrhosis requiring transplantation in the next decade. However, public awareness and perception of the disease are still extremely limited.

Therefore, our rationale is to evaluate the knowledge, attitudes, and determinants of NAFLD among adults in Jazan, Saudi Arabia. The main objectives of this study are to assess the public’s knowledge and attitudes regarding NAFLD and to identify demographic factors associated with these aspects. Conducting this study is essential for developing effective preventive strategies and interventions in the region.

## Materials and methods

Study design and participants

A cross-sectional study was conducted to estimate the knowledge, attitudes, and influencing factors regarding NAFLD among the general population in the Jazan region. The Jazan region, located in the southwestern part of Saudi Arabia, is one of the country’s 13 provinces [11]. Based on the 2022 census, Jazan has a population of over 1.4 million people [12]. This study focused on the adult Saudi population of Jazan, specifically those aged 18 and above who are fluent in both Arabic and English. Exclusion criteria included individuals who refused to be recruited in the study, nonresidents of Jazan, and those under 18 years of age. To gather data from the general population, participants were recruited from Jazan’s 17 provinces using a nonrandomized convenience sampling method. The sample size was calculated using Raosoft software (Raosoft Inc., Seattle, Washington, United States), aiming for a 95% confidence level and a 50% estimated response distribution with a ±5% margin of error. The minimum required sample size was determined to be 385 participants. However, the target sample size was increased to 560 participants [13].

Data collection tool

The study adopted a validated, self-administered online questionnaire to collect data. After obtaining permission, the questionnaire was developed from a similar study that aimed to assess the knowledge and attitudes of the Taif community regarding NAFLD [10]. The questionnaire comprised three sections with a total of 22 questions. The first one included 11 questions covering demographic information such as gender, age, education level, occupation, state of residence, marital status, and monthly family income in Saudi riyals. Additionally, it collected data on chronic diseases, height, and weight to calculate BMI. Participants were also asked whether any family member had been diagnosed with NAFLD to further assess their knowledge. The second part evaluated participants’ knowledge of NAFLD through four true or false questions, while the final part examined participants’ attitudes toward NAFLD, featuring seven true or false statements about the disease.

Data collection process and data analysis

The data collection process took place between November 2023 and January 2024 through various social media platforms (WhatsApp, Telegram, X, and Instagram). Participants were informed about the study’s objectives and their right to withdraw at any time. Following data collection, the responses were manually reviewed and coded into a Microsoft Excel sheet (Microsoft Corporation, Redmond, Washington, United States) before being entered into and analyzed with R software version 4.2.2 (The R Foundation, Vienna, Austria). The analysis involved both descriptive and comparative statistics. Descriptive statistics included frequencies and percentages for qualitative variables, as well as the mean and SD for quantitative variables. The chi-square test was used to identify associations between sociodemographic factors and levels of knowledge and attitudes toward NAFLD, with a p-value of less than 0.05 indicating statistical significance.

To evaluate participants’ knowledge of NAFLD, four questions were included in the questionnaire, in which participants were asked to find out whether they had heard about NAFLD before, whether they knew its high prevalence, and the risk it carries along with its dangerous nature. The last question was to find out whether the participant thinks that he or she is at risk of NAFLD. Correct answers were scored as one point, whereas incorrect answers were not awarded any points. A scoring system was developed and analyzed based on the total points achieved and then categorized into three groups: poor, average/fair, or good knowledge. Poor knowledge level is defined as scoring one or no correct answers out of the four questions, while average or fair knowledge level is defined as answering two or three questions correctly out of the four questions. A good knowledge level was defined as answering all the questions correctly. Like knowledge, attitudes toward NAFLD were assessed by asking the participants about certain aspects of NAFLD, such as their willingness to undergo medical screening for it and the comorbidities that lead to it, such as obesity, diabetes, low-density lipoprotein, and hypertension. Furthermore, items were also included to assess their attitude toward exercise involvement in avoiding NAFLD and the risk of cancer associated with NAFLD. The total score was derived from the points accumulated, and a scoring system was established to classify participants’ attitudes as negative if the total score equals 2 or fewer correct statements, and a neutral attitude is labeled for the participant if the total score equals 5 or fewer correct statements. A positive attitude was labeled for the participant if the total score equals 6 or more correct statements.

Ethical consideration

Ethical principles were considered when collecting and processing data, as approved by the Jazan University Scientific Research Ethics Committee (approval number REC-45/04/083). Participants were informed about the study objectives and their rights to participate or to reject participation at any time. Moreover, participants were informed about their full confidentiality.

## Results

A total of 540 participants from the Jazan region of Saudi Arabia completed the questionnaire, resulting in a 96.4% response rate. Of these participants, 311 (58%) were male and 229 (42%) were female. Among them, 229 (42%) were aged between 18 and 29 years. Educationally, 274 (51%) held a higher degree, and 154 (29%) were employed in non-healthcare fields. Geographically, 279 (52%) lived in rural areas, 324 (60%) were married, and 241 (45%) reported a family income of 10,000-19,999 Saudi riyals. In terms of BMI, over one-third of participants (n = 209; 39%) were in the healthy range, while 116 (21%) were classified as obese. Additionally, 436 (81%) reported having no chronic diseases, and approximately 496 (92%) had no family members diagnosed with NAFLD (Table 1).

**Table 1:** Demographic distribution of the participants The demographics of the participants are represented as frequencies (n) and percentages (%). NAFLD, nonalcoholic fatty liver disease

Demographics	Frequency and proportion, n (%)
Age	
18-29	229 (42.4%)
30-39	127 (23.5%)
40-49	129 (23.9%)
More than 50	55 (10.2%)
Gender	
Female	229 (42.4%)
Male	311 (57.6%)
Educational level	
Before university education	89 (16.5%)
Higher education	274 (50.7%)
University education	177 (32.8%)
Occupation	
Employed in a non-healthcare field	154 (28.6%)
Employed in the healthcare field	64 (11.9%)
University student majoring in a healthcare field	107 (19.9%)
University student majoring in a non-healthcare field	46 (8.5%)
Retired	9 (1.7%)
Not working	19 (3.5%)
Others	140 (26.0%)
Unknown	1
Residence	
Mountain area	10 (1.9%)
Rural	279 (51.7%)
Urban	251 (46.5%)
Marital status	
Married	324 (60%)
Unmarried	216 (40%)
Average monthly family income (in Saudi riyals)	
Less than 4,999	86 (15.9%)
5,000-9,999	117 (21.7%)
10,000-19,999	241 (44.6%)
More than 20,000	96 (17.8%)
BMI	
Underweight	48 (8.9%)
Normal weight	209 (38.7%)
Overweight	167 (30.9%)
Obese	116 (21.5%)
Chronic diseases	
Diabetes mellitus	36 (6.7%)
Hypertension	37 (6.9%)
I don’t have any chronic diseases	436 (80.7%)
Others	31 (5.7%)
Family history of NAFLD	
No	496 (91.9%)
Yes	44 (8.1%)

Table 2 demonstrates the knowledge elements of NAFLD. The majority of the participants (n = 345; 63.8%) reported having not heard about NAFLD before, and 172 (31.9%) believed that the prevalence of NAFLD in Saudi Arabia is high. When assessing the potential severity of NAFLD, 442 (81.9%) acknowledged its ability to cause dangerous conditions. In terms of personal risk perception, 362 (67.0%) felt they were not at risk of NAFLD, while 178 (32.9%) considered themselves at risk.

**Table 2:** Description of participants’ answers to knowledge items Participants’ answers to knowledge items are represented as frequencies (n) and percentages (%). NAFLD, nonalcoholic fatty liver disease

Question	No	Yes
Have you heard about NAFLD before?	345 (63.8%)	195 (36.1%)
Do you think the prevalence rate of NAFLD in Saudi Arabia is high?	368 (68.1%)	172 (31.9%)
Do you think NAFLD can cause dangerous conditions?	98 (18.1%)	442 (81.9%)
Do you think you are at risk of NAFLD?	362 (67.0%)	178 (32.9%)

Table 3 demonstrates attitude elements toward NAFLD. Regarding medical screening for NAFLD, a majority of the participants (n = 490; 90.7%) expressed willingness to undergo the screening. Moreover, 471 (87.2%) recognized the association between obesity and NAFLD, while 69 (12.9%) did not perceive obesity as a cause. Similarly, 370 (68.5%) believed that diabetes causes NAFLD, 456 (84.4%) believed that high blood cholesterol can cause NAFLD, and 364 (67.4%) believed in the influence of hypertension on NAFLD. Approximately 437 (80.9%) recognized the positive effect of exercise in preventing NAFLD, while 103 (19.1%) did not share this perspective. Additionally, 412 participants (76.3%) believed in the potential association between NAFLD and liver cancer.

**Table 3:** Description of participants’ answers to attitude items Participants’ answers to attitude items are represented as frequencies (n) and percentages (%). NAFLD, nonalcoholic fatty liver disease

Question	No	Yes
Would you undergo medical screening for NAFLD?	50 (9.3%)	490 (90.7%)
Do you think obesity causes NAFLD?	69 (12.9%)	471 (87.2%)
Do you think diabetes causes NAFLD?	170 (31.5%)	370 (68.5%)
Do you think high levels of blood cholesterol cause NAFLD?	84 (15.5%)	456 (84.4%)
Do you think hypertension affects NAFLD?	176 (32.6%)	364 (67.4%)
Do you think exercise affects the avoidance of NAFLD?	103 (19.1%)	437 (80.9%)
Do you think NAFLD can cause liver cancer?	128 (23.7%)	412 (76.3%)

**Figure 1:**
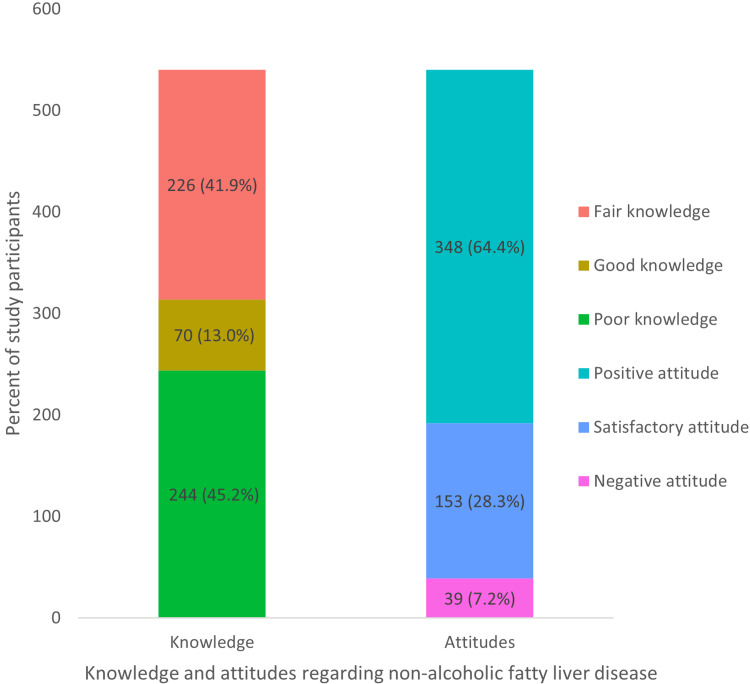
Description of the knowledge and attitude levels of participants toward NAFLD NAFLD, nonalcoholic fatty liver disease

Concerning knowledge and attitude levels regarding NAFLD, the majority of the participants (244, 45.2%) demonstrated poor knowledge of NAFLD, while 226 (41.9%) had fair knowledge. Additionally, 70 (13%) participants demonstrated good knowledge. In terms of attitudes, the overwhelming majority (n = 348; 64.4%) of the participants exhibited a positive attitude, and 28.3% (n = 153) of participants exhibited a satisfactory attitude. Only 7% (n = 39) of the sample showed a poor attitude regarding NAFLD (Figure 1).

While analyzing the sociodemographic factors associated with the knowledge of NAFLD, several factors were associated with the knowledge of NAFLD. Specifically, individuals aged 40-49, males, those employed in the healthcare sector, individuals with obesity and diabetes mellitus, and those with a family history of NAFLD exhibited significantly higher levels of knowledge about NAFLD (p < 0.05) (Table 4).

**Table 4:** Sociodemographic factors associated with levels of knowledge toward NAFLD The demographics of the participants are represented as frequencies (n) and percentages (%). The chi-square test was used to investigate statistical significance; a p-value of less than 0.05 is considered to be statistically significant. NAFLD, nonalcoholic fatty liver diseases

Demographics	Fair knowledge	Good knowledge	Poor knowledge	Chi-square value	p-value
Age				16.958	0.009
18-29	109 (48.2%)	22 (31.4%)	98 (40.2%)		
30-39	47 (20.8%)	14 (20.0%)	66 (27.0%)		
40-49	42 (18.6%)	26 (37.1%)	61 (25.0%)		
More than 50	28 (12.4%)	8 (11.4%)	19 (7.8%)		
Gender				9.186	0.01
Female	102 (45.1%)	18 (25.7%)	109 (44.7%)		
Male	124 (54.9%)	52 (74.3%)	135 (55.3%)		
Educational level				8.322	0.08
Before university education	32 (14.2%)	7 (10.0%)	50 (20.5%)		
University education	84 (37.2%)	22 (31.4%)	71 (29.1%)		
Higher education	110 (48.6%)	41 (58.6%)	123 (50.4%)		
Occupation				77.467	<0.001
Employed in a non-healthcare field	57 (25.2%)	17 (24.3%)	80 (32.9%)		
Employed in the healthcare field	32 (14.2%)	20 (28.6%)	12 (4.9%)		
University student majoring in a healthcare field	67 (29.6%)	16 (22.9%)	24 (9.9%)		
University student majoring in a non-healthcare field	15 (6.6%)	0 (0%)	31 (12.8%)		
Not working	7 (3.1%)	0 (0%)	12 (4.9%)		
Others	45 (19.9%)	16 (22.9%)	79 (32.5%)		
Retired	3 (1.3%)	1 (1.4%)	5 (2.1%)		
Unknown	0	0	1		
Residence				1.094	0.8
Mountain area	4 (1.8%)	2 (2.9%)	4 (1.6%)		
Rural	120 (53.1%)	33 (47.1%)	126 (51.6%)		
Urban	102 (45.1%)	35 (50.0%)	114 (46.7%)		
Marital status				4.713	0.095
Married	124 (54.9%)	47 (67.1%)	153 (62.7%)		
Unmarried	102 (45.1%)	23 (32.9%)	91 (37.3%)		
Average monthly family income (in Saudi riyals)				7.99	0.2
Less than 4,999	29 (12.8%)	12 (17.1%)	45 (18.4%)		
5,000-9,999	50 (22.1%)	9 (12.9%)	58 (23.8%)		
10,000-19,999	104 (46.0%)	33 (47.1%)	104 (42.6%)		
More than 20,000	43 (19.0%)	16 (22.9%)	37 (15.1%)		
BMI				21.566	0.001
Underweight	27 (11.9%)	0 (0%)	21 (8.6%)		
Normal weight	91 (40.3%)	23 (32.9%)	95 (38.9%)		
Overweight	57 (25.2%)	23 (32.9%)	87 (35.7%)		
Obese	51 (22.6%)	24 (34.3%)	41 (16.8%)		
Chronic diseases				24.188	0.002
Diabetes	14 (6.2%)	13 (18.6%)	9 (3.7%)		
Hypertension	17 (7.5%)	7 (10%)	13 (5.3%)		
I don’t have any chronic diseases	185 (81.9%)	45 (64.3%)	206 (84.4%)		
Others	10 (4.4%)	5 (7.1%)	16 (6.6%)		
Family history of NAFLD				67.342	<0.001
No	212 (93.8%)	47 (67.1%)	237 (97.1%)		
Yes	14 (6.2%)	23 (32.9%)	7 (2.9%)		

Similar to knowledge, the attitudes of the participants were analyzed to investigate the sociodemographic factors associated with the attitudes toward NAFLD. Education level was found to be the only factor influencing attitudes toward NAFLD, as individuals with higher education exhibited a more positive attitude compared to those with education below the university level (p = 0.004) (Table 5).

**Table 5:** Sociodemographic factors associated with levels of attitudes toward NAFLD The demographics of the participants are represented as frequencies (n) and percentages (%). The chi-square test was used to investigate statistical significance; a p-value of less than 0.05 is considered to be statistically significant. NAFLD, nonalcoholic fatty liver diseases

Demographics	Negative attitude	Positive attitude	Satisfactory attitude	Chi-square value	p-value
Age				3.084	0.8
18-29	20 (51.3%)	148 (42.5%)	61 (39.9%)		
30-39	6 (15.4%)	81 (23.3%)	40 (26.1%)		
40-49	8 (20.5%)	85 (24.4%)	36 (23.5%)		
More than 50	5 (12.8%)	34 (9.8%)	16 (10.5%)		
Gender				0.254	0.9
Female	18 (46.2%)	146 (42.0%)	65 (42.5%)		
Male	21 (53.8%)	202 (58.0%)	88 (57.5%)		
Educational level				15.285	0.004
Before university education	13 (33.3%)	52 (14.9%)	24 (15.7%)		
University education	13 (33.3%)	125 (35.9%)	39 (25.5%)		
Higher education	13 (33.3%)	171 (49.1%)	90 (58.8%)		
Occupation				17.556	0.066
Employed in a non-healthcare field	8 (20.5%)	97 (27.9%)	49 (32.2%)		
Employed in the healthcare field	6 (15.4%)	40 (11.5%)	18 (11.8%)		
Not working	2 (5.1%)	11 (3.2%)	6 (3.9%)		
Others	13 (33.3%)	83 (23.9%)	44 (28.9%)		
Retired	0 (0%)	5 (1.4%)	4 (2.6%)		
University student majoring in a healthcare field	6 (15.4%)	85 (24.4%)	16 (10.5%)		
University student majoring in a non-healthcare field	4 (10.3%)	27 (7.8%)	15 (9.9%)		
Unknown	0	0	1		
Residence				4.558	0.3
Mountain area	2 (5.1%)	5 (1.4%)	3 (2.0%)		
Rural	18 (46.2%)	175 (50.3%)	86 (56.2%)		
Urban	19 (48.7%)	168 (48.3%)	64 (41.8%)		
Marital status				1.446	0.5
Married	21 (53.8%)	206 (59.2%)	97 (63.4%)		
Unmarried	18 (46.2%)	142 (40.8%)	56 (36.6%)		
Average monthly family income (in Saudi riyals)				7.229	0.3
Less than 4,999	7 (17.9%)	47 (13.5%)	32 (20.9%)		
5,000-9,999	10 (25.6%)	71 (20.4%)	36 (23.5%)		
10,000-19,999	17 (43.6%)	162 (46.6%)	62 (40.5%)		
More than 20,000	5 (12.8%)	68 (19.5%)	23 (15.0%)		
BMI				3.667	0.7
Underweight	6 (15.4%)	30 (8.6%)	12 (7.8%)		
Normal weight	11 (28.2%)	138 (39.7%)	60 (39.2%)		
Overweight	13 (33.3%)	108 (31.0%)	46 (30.1%)		
Obese	9 (23.1%)	72 (20.7%)	35 (22.9%)		
Chronic diseases				2.498	0.9
Diabetes	3 (7.7%)	21 (6.0%)	12 (7.8%)		
Hypertension	1 (2.6%)	27 (7.8%)	9 (5.9%)		
I don’t have any chronic diseases	33 (84.6%)	279 (80.2%)	124 (81.0%)		
Others	2 (5.1%)	21 (6.0%)	8 (5.2%)		
Family history of NAFLD				1.779	0.5
No	37 (94.9%)	322 (92.5%)	137 (89.5%)		
Yes	2 (5.1%)	26 (7.5%)	16 (10.5%)		

## Discussion

Our study aimed to investigate the level of knowledge and attitudes toward NAFLD among adults in Jazan Province. We also aimed to identify the factors that influence these knowledge and attitudes. To our knowledge, this was the first attempt made in the region. Our findings revealed that while there is a moderate level of knowledge about NAFLD, certain misconceptions and gaps in knowledge appear in our final analysis. Furthermore, participants recognized the potential severity of NAFLD but had a low personal risk perception. In terms of attitudes regarding NAFLD, our participants expressed a positive attitude toward medical screening and demonstrated an understanding of the association between NAFLD and obesity, diabetes, high blood cholesterol, and hypertension, although some misconceptions existed. Certain demographic and health-related factors influenced both knowledge and attitudes about NAFLD, emphasizing the importance of targeted educational interventions.

In terms of the knowledge of the participants regarding NAFLD, almost two-thirds of the study participants reported having prior knowledge of NAFLD. This implies a reasonable grasp of NAFLD among the research population. In addition, our survey revealed that while a larger percentage of respondents (n = 226; 41.9%) had fair knowledge of NAFLD, just 13% (n = 70) of respondents had good knowledge of the illness overall. On the other hand, a lower percentage was found in a prior study among the Taif population in Saudi Arabia [10]. Similarly, low knowledge levels were also reported in various studies around the world. For instance, a previous survey of the general Saudi Arabian population found that 50% (n = 780) of respondents had fair knowledge and only 7% (n = 115) had high knowledge [14]. Insufficient knowledge of NAFLD was also reported by 83% (n = 433) of respondents in a study performed in Hong Kong [7]. NAFLD was not well known to most participants, irrespective of their demographics, according to a different US study [9].

Concerning the prevalence of NAFLD, a considerable fraction (n = 172; 31.9%) of the people who knew about it thought that it was quite prevalent in Saudi Arabia. This aligns with the growing literature in which NAFLD prevalence is on the rise [6]. This is also reflected in the majority of the participants due to their recognition of the potentially harmful complications that can result from NAFLD. Our analysis also revealed that significantly greater levels of NAFLD knowledge were found among several vulnerable groups, such as those with diabetes mellitus and obesity. Diabetes mellitus and obesity are well-known risk factors for the development of NAFLD [5]; this fact may lead diabetic and obese patients to educate themselves more about their health condition, resulting in their high knowledge of the complications of both diabetes mellitus and obesity. Males who are between 40 and 49 years old, healthcare workers, and those with a family history of NAFLD also showed greater knowledge levels. This emphasizes the significance of focused educational initiatives to raise NAFLD awareness among particular population categories, especially those who are more vulnerable.

In terms of the attitudes of the participants toward NAFLD, our study revealed various attitudes among participants concerning NAFLD and its risk factors. The findings indicated that a majority of participants (n = 490; 90.7%) expressed a willingness to undergo medical screening for NAFLD, reflecting a positive attitude toward a proactive approach to healthcare seeking in the case of NAFLD. This finding aligns with a study conducted in Singapore, where (n = 129; 73.7%) of participants reported their willingness to undergo medical screening for NAFLD. The similarity in these findings indicates a consistent trend of individuals recognizing the importance of early detection and diagnosis of NAFLD, regardless of the specific population [15]. Additionally, a significant percentage (n = 456; 84.4%) recognized the relationship between high blood cholesterol levels and NAFLD, while an overwhelming majority (n = 471; 87.2%) acknowledged the relationship between obesity and NAFLD. This actively indicates a positive attitude toward understanding the role of obesity and high levels of blood cholesterol as causes of NAFLD [16,17]. Similarly, our results align with a previous study conducted in the United States where a considerable number of participants recognized obesity, hypertriglyceridemia, and diabetes as significant risk factors for NAFLD, which is consistent with our findings. These results demonstrate a consistent understanding among participants that fat buildup in the liver serves as a risk factor for NAFLD [9].

However, despite the positive attitudes observed, the study also uncovered significant gaps in knowledge and awareness regarding NAFLD and its risk factors. Notably, a considerable proportion of participants (n = 170; 31.5%) did not recognize the relationship between diabetes and NAFLD, and a significant number (n = 176; 32.6%) were unaware of the relationship between hypertension and NAFLD. These findings align with the study conducted in Taif, Saudi Arabia, which reported that only half of the participants acknowledged the relationship between NAFLD and metabolic diseases [10]. It is well established that metabolic syndrome, which includes hypertension and diabetes, serves as a strong risk factor for the development of NAFLD. This chronic liver condition can be caused by a combination of factors, such as insulin resistance, compensatory hyperinsulinemia, central abdominal obesity, and a reduction in high-density lipoprotein cholesterol [18]. Therefore, considering the knowledge gap identified in our study regarding metabolic syndrome as a cause of NAFLD, along with the high prevalence of diabetes (16.4%) and hypertension (22.66%) among Saudis, individuals with metabolic syndrome are at higher risk of developing NAFLD [19,20]. This relationship explains the rise in the prevalence of NAFLD in Saudi Arabia, which is estimated to affect 12,534,000 Saudi individuals by the year 2030 [21]. Thus, it is crucial to prioritize both education and preventive measures such as screening those affected by metabolic syndrome, raising public awareness, encouraging lifestyle and dietary modifications, and emphasizing the importance of regular medical checkups.

In terms of preventive measures, the study yielded noteworthy findings. The majority of participants (n = 437; 80.9%) recognized the beneficial effects of exercise in preventing NAFLD. This awareness highlights the importance of promoting regular physical activity as a proactive measure to reduce the risk of developing NAFLD. Hence, promoting physical activity as a preventive measure could be effective in overcoming the burden of NAFLD. Furthermore, a significant proportion (n = 412; 76.3%) of participants acknowledged the potential association between NAFLD and liver cancer. This indicates a positive attitude toward understanding the long-term consequences and complications that can arise from NAFLD. In contrast, a study conducted in the United States revealed that 84% (n = 4,235) of individuals claimed to be unaware of the conditions that can potentially lead to liver cirrhosis [9]. This stark contrast reflects the difference between the two populations in terms of knowledge regarding the long-term consequences and complications that can arise from NAFLD. Thus, raising public awareness and understanding their attitudes is crucial to controlling NAFLD, as it poses serious threats to individuals and the healthcare system worldwide.

Strengths and limitations

This study has several strengths. One major advantage is its provision of valuable insights into the knowledge, attitudes, and determinants regarding NAFLD among adults in Jazan Province. The use of validated questions from a similar study [10] enhances the validity and reliability of the results. Additionally, the study addresses a crucial knowledge gap by illuminating the understanding and attitudes toward NAFLD in this region.

However, there are limitations. The convenience sampling technique may introduce selection bias, affecting the generalizability of the findings. As with most cross-sectional studies, it does not establish causality between the examined variables. The reliance on self-reported data may also introduce recall bias. Furthermore, the study did not explore the role of dietary factors and their association with NAFLD knowledge and attitudes, which is a potential area for future research. Future studies could consider longitudinal designs to improve robustness and include dietary factors to provide a more comprehensive understanding of the factors influencing NAFLD knowledge and attitudes.

## Conclusions

The study highlights the levels of knowledge and attitudes about NAFLD among adults in Jazan Province. While a significant portion of individuals demonstrated a fair understanding of NAFLD, most participants had poor knowledge. On a positive note, the majority displayed a positive attitude toward NAFLD, with 28.3% showing a satisfactory attitude. These findings underscore the need for targeted educational interventions and public awareness campaigns to enhance understanding of NAFLD in Jazan Province. Providing accurate and current information about NAFLD, its consequences, and preventive measures is crucial. Future research could also investigate the role of dietary factors and their relationship with NAFLD knowledge and attitudes.

## Appendices

**Table 6:** Study questionnaire

Section 1: Demographic information				
1. Age		18-29		
		30-39		
		40-49		
		More than 50		
2. Gender		Female		
		Male		
3. Level of education		Before university education		
		Higher education		
		University education		
4. Occupation		Employed in a non-healthcare field		
		Employed in the healthcare field		
		University student majoring in a healthcare field		
		University student majoring in a non-healthcare field		
		Retired		
		Not working		
		Other (please specify)		
5. State of residence		Mountain area		
		Rural		
		Urban		
6. Marital status		Married		
		Unmarried		
7. Average monthly family income		Less than 4,999SR		
		5,000-9,999SR		
		10,000-19,999SR		
		More than 20,000SR		
8. Chronic diseases		Diabetes mellitus		
		Hypertension		
		I don’t have any chronic diseases		
		Others (please specify)		
9. Weight in kg				
10. Height in cm				
11. Any family member diagnosed with NAFLD?		Yes		
		No		
Section 2: Knowledge toward NAFLD				
Item	Statement		Yes	No
1	Have you heard about NAFLD before today?			
2	Do you think the prevalence rate of NAFLD in Saudi Arabia is high?			
3	Do you think NAFLD can cause dangerous conditions?			
4	Do you think you are at risk of NAFLD?			
Section 3: Attitudes toward NAFLD				
Item	Statement		Yes	No
1	Would you undergo medical screening for NAFLD?			
2	Do you think obesity cause NAFLD?			
3	Do you think diabetes causes NAFLD?			
4	Do you think high levels of blood cholesterol cause NAFLD?			
5	Do you think hypertension affects NAFLD?			
6	Do you think exercise affect the avoidance of NAFLD?			
7	Do you think NAFLD can cause liver cancer?			
